# Advanced HIV Disease at Enrolment in HIV Care: Trends and Associated Factors over a Ten Year Period in Cambodia

**DOI:** 10.1371/journal.pone.0143320

**Published:** 2015-11-25

**Authors:** Reaksmey Pe, Bopha Chim, Sopheak Thai, Lutgarde Lynen, Johan van Griensven

**Affiliations:** 1 Sihanouk Hospital Center of HOPE, Phnom Penh, Cambodia; 2 Institute of Tropical Medicine, Antwerp, Belgium; University of New South Wales, AUSTRALIA

## Abstract

**Background:**

Early HIV diagnosis and enrolment in care is needed to achieve early antiretroviral treatment (ART) initiation. Studies on HIV disease stage at enrolment in care from Asian countries are limited. We evaluated trends in and factors associated with late HIV disease presentation over a ten-year period in the largest ART center in Cambodia.

**Methods:**

We conducted a retrospective analysis of program data including all ARV-naïve adults (> 18 years old) enrolling into HIV care from March 2003-December 2013 in a non-governmental hospital in Phnom Penh, Cambodia. We calculated the proportion presenting with advanced stage HIV disease (WHO clinical stage IV or CD4 cell count <100 cells/μL) and the probability of ART initiation by six months after enrolment. Factors associated with late presentation were determined using multivariate logistic regression.

**Results:**

From 2003–2013, a total of 5642 HIV-infected patients enrolled in HIV care. The proportion of late presenters decreased from 67% in 2003 to 44% in 2009 and 41% in 2013; a temporary increase to 52% occurred in 2011 coinciding with logistical/budgetary constraints at the national program level. Median CD4 counts increased from 32 cells/μL (IQR 11–127) in 2003 to 239 cells/μL (IQR 63–291) in 2013. Older age and male sex were associated with late presentation across the ten-year period. The probability of ART initiation by six months after enrolment increased from 22.6% in 2003–2006 to 79.9% in 2011–2013.

**Conclusion:**

Although a gradual improvement was observed over time, a large proportion of patients still enroll late, particularly older or male patients. Interventions to achieve early HIV testing and efficient linkage to care are warranted.

## Background

There are currently an estimated 35 million individuals living with the human immunodeficiency virus (HIV) at the global level [1]. Of these, close to 5 million live in Asia. Over the last decade, an impressive scaling-up of antiretroviral treatment (ART) has occurred, including in low and middle income countries, with currently over ten million people on treatment [1].

Since starting ART with advanced disease is associated with higher mortality and morbidity, and missed opportunities for HIV prevention, ART needs to be started early to have maximal impact [2,3]. The World Health Organization (WHO) guidelines have gradually increased the CD4 cell count threshold for ART initiation, with a cut-off of 500 cells/μL proposed in the 2013 version [4]. However, the effect of these changes highly depends on whether HIV patients can be diagnosed early, with subsequent enrolment and retention into care. A recent multi-country study from Africa demonstrated that advanced HIV disease—defined as a WHO clinical stage IV or a CD4 cell count < 100 cells/μL—at the time of HIV diagnosis remains common [5]. Men were particularly likely to present late. Studies have also demonstrated that late presentation remains a problem in high income countries such as the United States of America and within Europe [6–8].

Late presentation is not only associated with higher mortality, but also with increased costs for the health system and increased HIV transmission [9,10].

Despite the substantial HIV burden, including in low-income countries, studies on this topic from Asia are surprisingly scarce, particularly from South-East Asia which is among the most affected Asian regions. Within South-East Asia, we only found one publication on late HIV presentation from Thailand, but this study reported on data from more than eight years ago [11].

Cambodia is a low income country in South-East Asia, initially among the most HIV affected countries in Asia. Reportedly, universal coverage of ART (>80% of eligible patients receiving ART) was achieved in 2011 [12]. The country now strives to be amongst the first countries to achieve “HIV elimination”. There have been some recent large multi-country studies on various aspect of HIV care using pooled data from numerous countries including also Cambodia and Thailand. However, these studies typically included few patients from South-East Asian countries, basically aimed to provide global estimates and, most importantly, focused on other operational challenges such as advanced HIV disease at ART initiation [13,14]. Using carefully collected program data, the aim of this study was to evaluate trends in and factors associated with advanced HIV disease at enrolment over a ten year period in the largest ART center in Cambodia.

## Methods

### Study design

We conducted a retrospective cohort study using routinely collected data.

### Study setting

Cambodia has an estimated population of 13 million inhabitants. While HIV prevalence reached 2.4% in 1998, the situation has improved dramatically with the prevalence estimated at less than 0.8% in 2008 [15]. There are currently almost 50.000 individuals on ART [12]. The Sihanouk Hospital Center of Hope (SHCH) in Phnom Penh, Cambodia, is a non-governmental hospital provides free comprehensive HIV care as part of the national ART program since March 2003. As the program is accessible to any individual irrespective of residence, the HIV cohort comprises individuals from throughout the country, with around half originating from outside the capital.

### Study population

All ARV-naïve adults (inpatients or outpatients; > 18 years old) enrolling in HIV care at the SHCH program between March 2003 and December 2013 were included. Those that had started ART in another center and subsequently presenting at SHCH (transfer-ins) were excluded. Individuals missing information on both WHO stage and CD4 count at enrolment—precluding the determination of the study outcome (advanced HIV disease at enrolment)—were excluded as well.

### HIV testing, enrolment into HIV care and ART initiation

There were different entry points for enrolment into HIV care at SHCH. In all SHCH hospital services, provider initiated testing and counselling (PITC) was applied. Testing was also proposed to partners of individuals enrolled in HIV care at SHCH. In addition, patients could be diagnosed outside of SHCH but (self-)referred for HIV care, either coming from a voluntary counselling and testing (VCT) service, either from another health facility/HIV care center (defined as “other” in the rest of the manuscript). All HIV testing and counselling within the hospital was done by HIV program staff. Unless referral to another program was preferred, individuals testing positive were enrolled the same day in the HIV program.

All newly enrolled HIV-positive patients were clinically staged upon enrolment, evaluated for ongoing opportunistic infections, and had a CD4 cell count done (FACSCount, Becton Dickinson, Franklin Lakes, NJ). Indications for ART followed WHO recommendations [16,17]. Pre-ART laboratory investigations included hematology, liver and renal function tests and hepatitis B/C evaluation. Patients not presenting at their scheduled visits were traced by home visits or by phone, and considered lost to follow-up (LTFU) if they were not found within six months after their most recent visit. Additional details of the ART program at SHCH have been published before [18–23].

### Outcomes and operational definitions

Advanced HIV disease at enrolment was defined as presenting with a CD4 cell count < 100 cells/μL or a WHO clinical stage IV, irrespective of CD4 counts, at enrolment into HIV care, in line with a recent multi-country study from Africa [5]. In the text, this is also referred to as “late presentation”.

The ten year period was divided into three periods: 2003–2006 (the early phase of the program and scaling-up), 2007–2010 (maturing of the program) and 2011–2013 (a period of budgetary and operational constraints for HIV care at the national level). By 2011, several international non-governmental organisations involved in HIV care handed over their activities. Combined with some budgetary constraints at the national level, this was also the time that programs on some occasions re-started restricting enrolment of new patients.

### Data collection and statistical analysis

At the HIV/ART program onset, structured clinical records, data collection tools, and a database were developed. On a daily basis, clinical, laboratory and treatment data were collected and electronically stored. Physicians were trained in standardized patient assessment using the hospital guidelines and protocols. Quality control of the stored data was performed at regular intervals.

The main focus of the analysis was on trends in late presentation and associated factors over the ten year period, stratified by sex. Medians (interquartile range (IQR)) and frequencies (%) were used to describe patients’ characteristics. Factors associated with advanced HIV disease at enrolment over the different periods were determined using logistic regression including the following *a priori* defined factors: age, sex and referral source. The proportion started on ART at three and six months after enrolment was calculated as well, accounting for the competing risks of death and LTFU [24,25]. Person-time at risk was calculated, starting from the date of enrolment in HIV care up to either the date of ART initiation, death, date of last visit for those LTFU or transferred-out, and 21 April 2014 for the remainder.

### Ethical issues

Since the launching of the HIV care program, clinical data have been routinely collected for purposes of program monitoring and evaluation, and research activities. Patients were requested to give informed consent to store and use the data. Data collection and informed consent procedures were approved by the Institutional Review Board of the SHCH and the Institute of Tropical Medicine, Antwerp, Belgium. No patient identifiers were included in the dataset used for this analysis. All patients included in the study gave written informed consent for their clinical records to be used in this study.

## Results

Over the ten year period, a total of 5723 ARV-naïve adults enrolled in the HIV program. With 81 individuals excluded for missing information on both baseline CD4 count and WHO clinical stage at enrolment, 5642 were included in analysis. The median age at enrolment was 34 (IQR 29–41) years, 47% were male. Most (62%; n = 3480) were referred from a VCT service outside the hospital, 28% were diagnosed with HIV in the hospital.

Patient characteristics stratified by period are presented in Table 1. The male-female ratio remained fairly stable over time. There was a slight increase in age over time, with a clear increase in the proportion of older (> 40 years) individuals increasing from 24% in 2003–2006 to 38% in 2011–2013. The median baseline CD4 count and the proportion presenting with WHO clinical stage I/II gradually increased over time.

**Table 1:** Characteristics of adults enrolling into HIV care stratified by period, Phnom Penh, Cambodia (2003–2013; N = 5642).

	2003–2006	2007–2010	2011–2013	*P* value
Total	2687	2188	767	
Age (years)				
Median (IQR)	34 (29–40)	34 (29–41)	36 (31–43)	<0.001
≤25; n (%)	257 (10%)	195 (9%)	56 (7%)	<0.001
26–30; n (%)	551 (21%)	481 (22%)	100 (13%)	
31–35; n (%)	698 (26%)	486 (22%)	205 (27%)	
36–40; n (%)	546 (20%)	420 (19%)	119 (16%)	
41–50; n (%)	478 (18%)	442 (20%)	198 (26%)	
>50; n (%)	157 (6%)	164 (8%)	89 (12%)	
Male sex; n (%)	1326 (49%)	986 (45%)	344 (45%)	0.005
Enrolment referral source; n (%) (n = 5620)				<0.001
Hospital	938 (35%)	459 (21%)	159 (21%)	
VCT	1271 (48%)	1643 (75%)	566 (74%)	
Other	460 (17%)	86 (4%)	33 (5%)	
WHO clinical stage (n = 5608)				<0.001
Stage I/II; n (%)	616 (23%)	823 (33%)	241 (31%)	
Stage III/IV; n (%)	2038 (77%)	1364 (62%)	526 (69%)	
Baseline CD4 count (cell/μL); median (IQR) (n = 4465)	84 (21–275)	145 (39–315)	164 (39–351)	<0.001
Late presenters; n (%)	1439 (53.5)	976 (44.6)	365 (47.6)	<0.001

IQR: interquartile range; VCT: voluntary counselling and testing; WHO: World Health Organization

**Fig 1:**
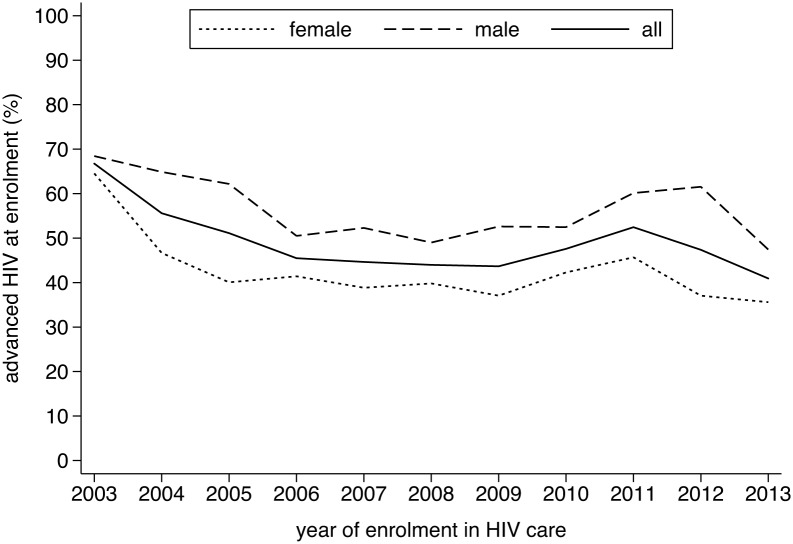
Evolution in the proportion of individuals enrolling with advanced HIV disease (CD4 cell count < 100 cells/μL or WHO clinical stage IV), Phnom Penh, Cambodia (2003–2013).

**Fig 2:**
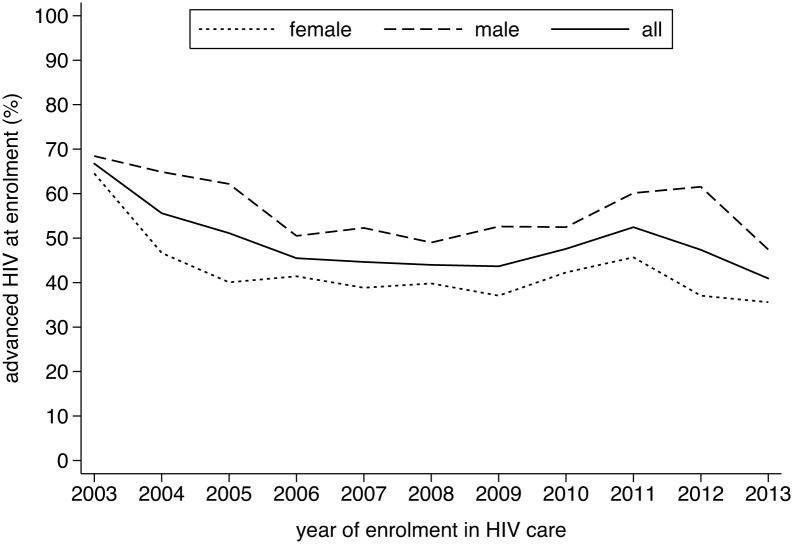
Evolution in the proportion of individuals enrolling with WHO clinical stage IV, Phnom Penh, Cambodia (2003–2013).

**Fig 3:**
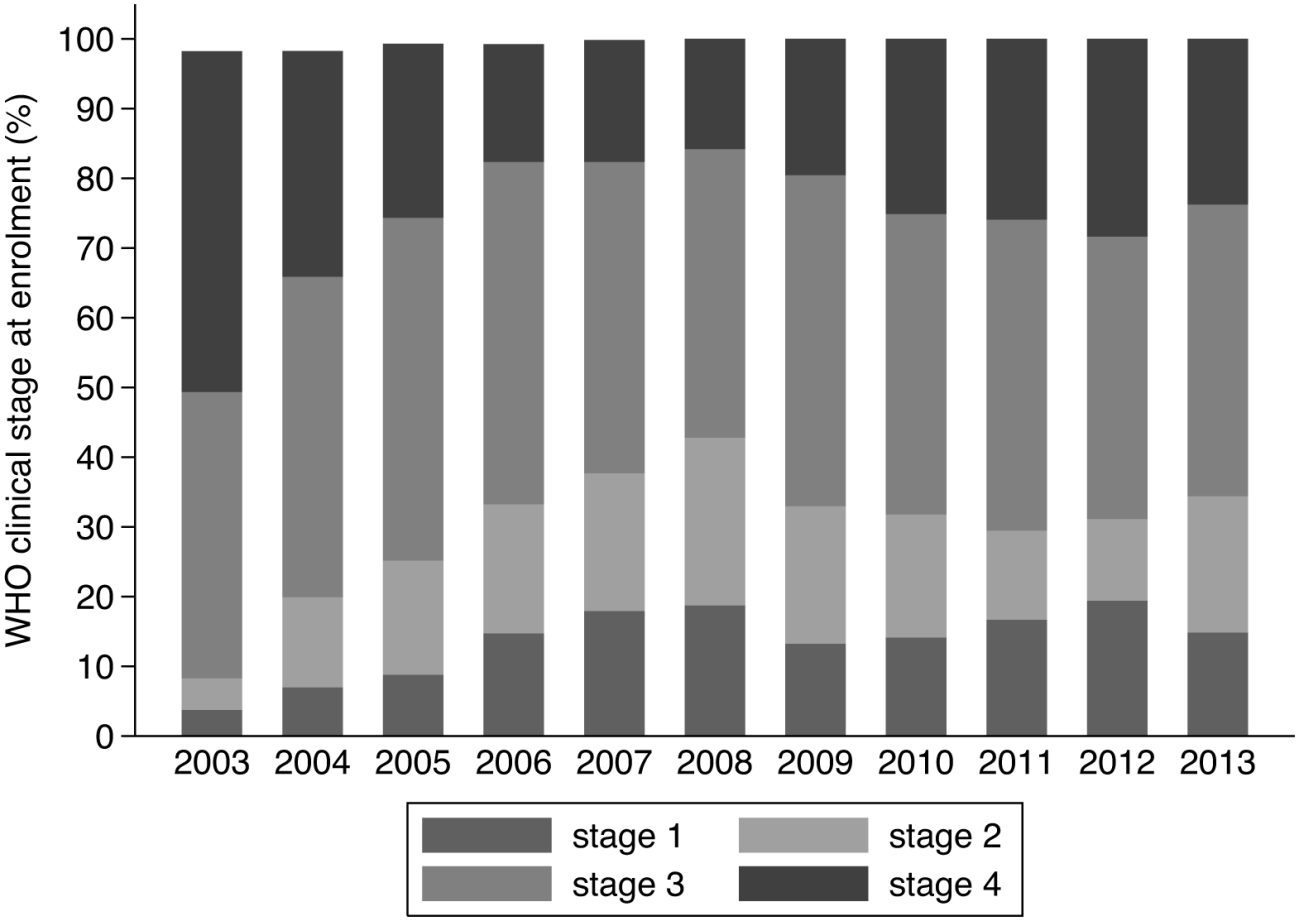
Evolution in the median CD4 count at enrolment into HIV care, Phnom Penh, Cambodia (2003–2013).

Overall, there were 2780 (49%) individuals presenting with advanced HIV diseases. As can be seen in Fig 1, the proportion of late presenters gradually decreased from 67% in 2003, with a temporary increase to 52% in 2011, to reach 41% in 2013. A similar pattern was observed in the median CD4 cell counts (Fig 2) and in the proportion presenting with WHO clinical stage IV (Fig 3). Across the different periods, the risk of presenting late was 40 to 80 percent higher for males (Table 2). Similarly, older age was positively associated with late presentation. Individuals referred from a VCT service were less likely to be late presenters.

**Table 2:** Factors associated with advanced HIV at enrolment in HIV care, Phnom Penh, Cambodia (2003–2013).

	2003–2006^a^	P value	2007–2010^a^	P value	2011–2013^a^	P value
	Adjusted OR (95% CI)		Adjusted OR (95% CI)		Adjusted OR (95% CI)	
Age (years)^b^ ^,^ ^c^						
≤25	1		1		1	
26–30	1.17 (0.87–1.60)	0.29	**2.09 (1.42–3.10)**	**<0.001**	1.84 (0.85–3.97)	0.12
31–35	**1.49 (1.11–2.02)**	**0.008**	**3.25 (2.21–4.80)**	**<0.001**	**2.45 (1.21–4.99)**	**0.013**
36–40	1.22 (0.90–1.66)	0.19	**3.48 (2.35–5.18)**	**<0.001**	**3.67 (1.74–7.74)**	**0.001**
41–50	**1.41 (1.03–1.94)**	**0.033**	**3.14(2.12–4.65)**	**<0.001**	**4.09 (2.00–8.33)**	**<0.001**
>50	1.13 (0.75–1.71)	0.55	**2.85 (1.79–4.54)**	**<0.001**	**2.88 (1.32–6.3)**	**0.008**
Sex						
Female	1		1		1	
Male	**1.83 (1.55–2.14)**	**<0.001**	**1.45 (1.22–1.74)**	**<0.001**	**1.75 (1.29–2.37)**	**<0.001**
Enrolment referral source^d^						
Hospital	1		1		1	
VCT	**0.70 (0.44–0.63)**	**<0.001**	**0.70 (0.57–0.87)**	**<0.001**	0.82 (0.56–1.18)	0.29
Other	0.86 (0.68–1.07)	0.19	0.68 (0.42–1.10)	0.12	2.0 (0.93–4.39)	0.077

CI: confidence interval; OR: adjusted odds ratio (adjusted for all variables in the table); VCT: voluntary counselling and testing

^a^Data are shown stratified per period since there were statistically significant interactions between period and age group, and between period and enrolment referral source.

Associations with a *P*-value < 0.05 are written in bold

^b^Overall P value for age: (2003–2006); <0.001 (2007–2010); <0.001 (2011–2013)

^c^P value for trend for age: 0.231 (2003–2006); <0.001 (2007–2010); <0.001 (2011–2013)

^d^Overall P value for enrolment referral source: <0.001 (2003–2006); 0.0043 (2007–2010); 0.032 (2011–2013)

During the period 2003–2006, the proportion who had died by 3 months was 4.3% (115/2687) and 6.8% (183/2687) by six months. For the same period, the proportion who were lost to follow-up by 3 months was 22.0% (592/2687) and 29.1% (781/2687) by six months.

Between 2007 and 2010, the proportion who had died by 3 months was 3.8% (83/2188) and 5.1% (111/2188) by six months. For the same period, the proportion who were lost to follow-up by 3 months was 15.6% (342/2188) and 18.9% (413/2188) by six months.

Between 2011 and 2013, the proportion who had died by 3 months was 7.9% (61/767) and 8.6% (66/767) by six months. For the same period, the proportion who were lost to follow-up by 3 months was 9.4% (72/767) and 12.0% (92/767) by six months.

During the 2003–2006 period, the estimated probability of ART initiation was 4.6% (95% confidence interval (CI) 3.5–5.9) by three months after enrolment and 22.6% (95% CI 20.2–25.0) by six months. This increased over the 2007–2010 period to 60.9% (95% CI 57.6–64.0) by three months and 77.2% (95% CI 74.4–79.8) by six months. For the most recent period (2011–2013), the corresponding values were 72.0% (95% CI 67.9–75.6) by three months and 79.9% (95% CI 76.2–83.2) by six months.

## Discussion

While the latest WHO ART guideline reinforces early initiation of ART, the programmatic impact of this guideline highly depends on early diagnosis and enrolment into HIV care [3]. Although we observed a gradual improvement over the ten year period, currently two out of every five new cases still present with advanced HIV disease. Males and older people were at particular high risk.

The 41% of enrolment with advanced disease in 2013 is higher than the 29% reported for 2011 in the recent multicenter study from sub-Saharan Africa [5]. However, the estimate in that study varied widely, from 14% in Rwanda to 41% in Tanzania. Moreover, data mainly originated from health centers. There are few studies from Asian countries that have specifically reported on disease status at HIV enrolment. A study from India found that 33% of individuals presented with a CD4 cell count below 200 cells/μL and 10% with a CD4 cell count below 50 cells/μL [26]. As to ART initiation, a recent Asian multi-country studies indicated that a substantial amount of patients still initiate ART with advanced disease [13].

Concurrent with the country-wide scaling-up of VCT services, the proportion of patients enrolling in our HIV care program via VCT services increased. While good access to HIV testing services is obviously key, an increase in late presentation was observed over the most recent period. This emphasizes that HIV testing is indeed only the first step in a complex cascade with retention on ART as endpoint and encompassing many steps such as enrolment in HIV care and retention in pre-ART care [27]. In this respect, the gradual increase over the ten year period in ART initiation by six months after enrolment in our study is encouraging.

Whereas the risk of delayed presentation for males has been well documented in older studies, it is concerning that this gap in (timely) access to HIV care remained high even up to the most recent period. The same was observed in a recent multi-country study in Africa [5] and in a study from India [26]. One would have hoped that with the scaling—up and decentralization of HIV/ART services and the increased global awareness of HIV (possibly also reducing stigma), sex disparities would have become attenuated. As suggested before, this gender difference could be due to many factors, including health care seeking behaviour, and the scaling-up and decentralisation of prevention of mother to child transmission (PMTCT) services [28–31].

Another finding of interest was the gradual increase in the proportion of older people enrolling into HIV care, who were also more likely to present with advanced clinical disease. Several other reports have raised the issue of old age and HIV in low and middle income countries, although mainly focussing on patients getting older while on ART and reporting data from Africa [32]. However, a study from China reported on this problem recently [33]. Why older individuals are also more likely to present late requires further study. It could for instance be that HIV testing is less likely to be proposed by the health care provider to older individuals, assuming the risk to be low. If this is confirmed, increased awareness among health care workers would be required. Conversely, the role out of and decentralisation of PMTCT services could facilitate early diagnosis for young mothers and their partners [34] and greater awareness.

The clear increase in late presentation in our program concurrent with reduced access to HIV care at the national level in from 2011 on could be due to many factors. A likely scenario is that—as a hospital providing free care for the poor—these individuals turned to our hospital as a last resort, after failing to achieve enrolment into HIV care in other health care facilities or after running out of cash. While ART and CD4 cell counts are provided free of charge within the national program, treatment and care for opportunistic infections has to be covered by the patient. Such events show that even successful national HIV programs remain vulnerable. Barriers to care (within the public sector) can easily re-appear and the most sick individuals and poor are the most likely to be affected. Late presentation has consequences beyond higher mortality, such as increased costs for the health system and increased HIV transmission [9,10].

While there remained a substantial burden of late presentation, it needs to be emphasized that the absolute number of new HIV enrolments has declined markedly concurrent with the national scaling-up of ART, demonstrating the overall success of the national program. Nevertheless, even in the era of universal access to ART, there remains a need for access to quality care for opportunistic infections, largely determined by late HIV diagnosis and late ART initiation. With a growing proportion of patients starting to fail second line treatment [35–39], and the currently limited access to third line regimens, the global burden of opportunistic infections might remain substantial for the next years to come.

There are a number of important limitations to this study. Clearly, our patient cohort does not represent a nationally representative sample. Moreover, as a hospital, the program is more likely to enroll sick individuals seeking care. For this reason, the main emphasis in this paper was on trends over time and less so on the absolute number of late presenters. While using the national program data would obviously have been beneficial, these also have a number of limitations (besides availability and quality). For instance, patients failing to enroll within the public health facilities might simply not feature in the statistics. As the only hospital consistently providing free care over the ten year period, and drawing patients from across the country, we feel our cohort somehow allows monitoring what occurs at the larger level. We also did not have information on other important risk factors for late presentation such as the mode of exposure, reasons to be tested for HIV, having a positive test for sexually transmitted infections and drug use. Detailed evaluation of socio-economic factors, including qualitative research, would have been highly useful to better understand the underlying barriers and reason for late presentation.

In conclusion, while observing a gradual improvement over the ten year period, around two out of five individuals currently still enroll with advanced HIV in our program. Male sex and older age were identified as risk factors. A temporary increase in late presentation was seen concurrent with a period of decreased access to HIV care at the national level. This demonstrates the vulnerability of HIV care programs, with those most in need of care (the sickest and the poorest) apparently most likely to be affected. Pending efforts to encourage early HIV diagnosis bear fruits, a substantial proportion of new individuals will continue to present late. Even in the era of universal access to ART, national programs should still ensure (access to) quality care for opportunistic infections.
